# Expand and Shrink: Federated Learning with Unlabeled Data Using Clustering

**DOI:** 10.3390/s23239404

**Published:** 2023-11-25

**Authors:** Ajit Kumar, Ankit Kumar Singh, Syed Saqib Ali, Bong Jun Choi

**Affiliations:** School of Computer Science and Engineering, Soongsil University, Seoul 06978, Republic of Korea; ajitkumar.pu@gmail.com (A.K.); aks.bihta@gmail.com (A.K.S.); saqib.kazmi149@gmail.com (S.S.A.)

**Keywords:** federated learning, unlabeled dataset, labeling, clustering, privacy preservation, deep learning, Internet of Things, supervised learning, weak supervision, semi-supervised learning

## Abstract

The amalgamation of the Internet of Things (IoT) and federated learning (FL) is leading the next generation of data usage due to the possibility of deep learning with data privacy preservation. The FL architecture currently assumes labeled data samples from a client for supervised classification, which is unrealistic. Most research works in the literature focus on local training, update receiving, and global model updates. However, by principle, the labeling must be performed on the client side because the data samples cannot leave the source under the FL principle. In the literature, a few works have proposed methods for unlabeled data for FL using “class-prior probabilities” or “pseudo-labeling”. However, these methods make either unrealistic or uncommon assumptions, such as knowing class-prior probabilities are impractical or unavailable for each classification task and even more challenging in the IoT ecosystem. Considering these limitations, we explored the possibility of performing federated learning with unlabeled data by providing a clustering-based method of labeling the sample before training or federation. The proposed work will be suitable for every type of classification task. We performed different experiments on the client by varying the labeled data ratio, the number of clusters, and the client participation ratio. We achieved accuracy rates of 87% and 90% by using 0.01 and 0.03 of the truth labels, respectively.

## 1. Introduction

Today, almost every digital solution is rapidly adopting artificial intelligence (AI) for products and services. These AI solutions are data-intensive, which result in exponential growth in data generation and sharing. The use of data for AI and Machine Learning (ML) is a privacy concern, and recently, many privacy-preserving solutions have been developed [1,2,3]. Federated learning (FL) is a potential solution gaining the attraction of researchers for privacy-preserving ML and deep learning (DL) [4,5]. Considering these, the Internet of Things (IoT) is becoming a key adopter of federated learning due to privacy preservation and restrictions on data movement from the source devices [6,7,8,9]. For example, Rahman et al. [8] discussed the application of FL for IoT intrusion detection by using the NSL-KDD dataset [10]. Similarly, there are other use cases for FL application in IoT. A plethora of academic and industrial researches have been carried out recently to produce software solutions and frameworks, that would assist the real world application of FL [11,12,13]. FL limits data sharing, i.e., raw data do not leave the client (restricted to generation point/source) system to be used for the training model. FL is derived from parallel and distributed computing and enables a model’s training in a federated manner by utilizing data from multiple clients. Currently, most FL algorithms are centered around supervised learning, where labeled data are assumed to be present on the client side. This requirement is achievable in a cross-silo (organizations act as participants and may have a data labeling team) setup. However, it is difficult to achieve in a cross-device setup due to several reasons, such as the cost, skills, and complexity associated with manual labeling by clients, as well as the large volume of data and the need for active participation.

In an actual or practical situation for cross-device FL, data at the client device are primarily unlabeled. For example, suppose pictures are stored in the client system for an object detection model. These pictures may be generated using the device camera, or the user can download them. Clearly, these pictures cannot always be assumed to be labeled and will not be ready for training. The data labeling issue for models that use medical imaging for training and testing was presented in [14].

The proposed work is inspired by weak supervision and semi-supervised learning and applies a clustering-based approach to label data at the client device. We assumed that the parametric server has a set of labeled data for the specific task (it is a valid assumption because the server needs to have labeled data for validating and tuning the global model). The client can seed the labeling process using a small fraction of these labeled data (shared once by the server with each client after joining the federation). In the proposed Expand and Shrink labeling approach, this tiny fraction of data (truth set) is mixed with the client data during the expand phase, and then clustering is applied. During the shrink phase, a set of clusters is shrunk to a class by comparing and tracing the clusters using the truth set. Labeling is performed once at the client in the current experiment, assuming that the client participates with the available data. However, the same approach can be repeated for new data (by mixing with previously labeled data or separately on new data).

Any FL averaging and communication strategy can be applied for post labeling at client side, therefore the proposed work is appropriate for the current FL architecture and federated averaging. The proposed work is suitable and required for using many devices available under the IoT because these devices generate lots of data, and often, these data need to have class labels. The proposed method uses a clustering-based approach that will make it easy to assign new query data, i.e., a sample to a particular cluster, and will have minimal cost. The proposed work is also compatible with existing model compression and quantization techniques, so a compressed model can be used instead of a large model for training IoT devices.

We performed different experiments on the client by varying the labeled data ratio, the number of clusters, and the client participation ratio. We obtained accuracy rates of 87% and 90% by using 0.01 and 0.03 of the truth labels, respectively. In general, with the proposed work, we made the following contributions:

- We proposed a data labeling method at the client device for supervised federated learning. The proposed labeling adopts pre-initialized centroid clustering methods to infer the class label of the unlabeled sample at the client device.
- We proposed an aggregation-independent labeling method that complements the existing supervised federated learning architecture, so no further changes are required in the existing communication and aggregation methods.
- We proposed a low cost in terms of the time and extensible labeling approach, i.e., a new sample can be labeled with reduced cost due to newly labeled samples.
- We performed extensive experiments to validate the proposed labeling method. In the federated learning setup, the proposed method provides equivalent performance to the human-labeled dataset in terms of accuracy. It achieves a similar level of global accuracy compared to the existing works while requiring much fewer truth labels.

The remainder of the paper is organized as follows. Section 2 presents existing work that deals with unlabeled data. Section 3 presents the proposed Expand and Shrink algorithm that employs a clustering-based approach to enable federated learning with unlabeled data. Section 4 compares the performance of the proposed algorithm with varying degrees of truth label availability. Our results show that the Expand and Shrink algorithm provides minimal labeling cost in terms of time and is extensible, thus allowing the labeling of a new sample at a lower cost. We conclude the paper in Section 5.

## 2. Related Work

In the supervised learning approach, unlabeled data are samples without any class label. Although the same data can have different class tags per the classification problem, the unlabeled data sample needs a label for the underlying classification task, for example, class labels between 0 and 9 for digit classification, such as in the MNIST dataset [15]. In general, the training set for supervised learning is indicated by ($x_{i},y_{i}$), where $x_{i}$ is a feature, and $y_{i}$ is the class label of the $i^{th}$ sample. The proposed work aims to assign $y_{i}^{\prime }$ for $x_{i}$, where $y_{i}^{\prime }$ is a noisy label and can be used for supervised training in the absence of the actual label during FL. There are many approaches to labeling datasets for supervised learning. In the semi-supervised method, some labeled data are used to annotate unlabeled data. Supervised training is carried out on the complete dataset (pre and post-labeled data sample). Semi-supervised learning has a smoothness assumption that if two samples *x* and $x^{\prime }$ are close in the input space, then their labels *y* and $y^{\prime }$ should be the same, i.e., d=(x,y) and $d^{\prime }=\left(x^{\prime },y^{\prime }\right)$. Building a supervised classifier using labeled and unlabeled data is familiar, and much literature is available on centralized machine learning.

Virginia R. de Sa [16] used structure between the pattern distributions of different sensory modalities to propose building a neural network (NN) model from unlabeled data. Caron et al. [17] used k-mean to cluster the features and then used cluster labels to update the NN weight during training. Recently, Jin et al. [18] adopted semi-supervised learning for federated learning to address the labeling task at the client. In semi-supervised learning, unlabeled data may degrade the model’s performance. Using federated learning, Albaseer et al. [19] applied semi-supervised learning for labeling and building a traffic sign detection model. Jeong et al. [20] used semi-supervised learning in two distinct scenarios: (a) labels-at-client (both labeled and unlabeled data are available at the client) and (b) label-at-server (labeled data are only with the server). Long et al. [21] also considered the label-at-server scenario and proposed FedCon, i.e., a contrastive learning-based federated learning framework. Rafa et al. [22] applied federated semi-supervised learning (FSSL) for Android malware detection; similarly, Pei et al. [23] applied transfer and semi-supervised learning with FL for IoT malware. Itahara et al. [24] used distillation-based semi-supervised FL to improve the communication for non independent and identically distributed (non-iid) data. Lu et al. [25] proposed FedUL, which assumes the availability of user class-conditional distributions, and used it to recover the required model from the global model each client trains with the help of surrogate labels for unlabeled data. Wang et al. [26] explored various setups to improve the semi-supervised federated learning (SSFL) performance and suggested that reducing gradient diversity can result in a fast and improved model. Zhu et al. [27] proposed to generate pseudo labels for unlabeled data using unlabeled data and global models. In each round, a temporary global model was trained that was tuned using the initial global model to obtain the final global model.

There was a recent development in semi-supervised learning, i.e., self-supervised learning (SSL), which removes the requirement of a human-annotated initial dataset to initiate semi-supervised learning. He et al. [28] used self-supervised learning for label deficiency in federated learning and also provided personalization for the client in FL. Yan et al. [29] used self-supervised federated learning to address the data heterogeneity and label deficiency in the medical domain (dataset retinal images, dermatology images, and chest X-rays). Wang et al. [30] also used contrastive visual representation learning and SSL for various tasks and studied the impact of non-iid and unlabeled data in FL. With the model-assisted labeling process, a small portion of the data is labeled to build an initial model that can be further used for only labeling, i.e., predicting labels for the remaining unlabeled data. An active learning-based FL approach was discussed, involving an oracle initially labeling a few unlabeled data at the client device [31].

There have been many modern approaches to centralized supervised learning for learning from unlabeled data, like transfer learning and few-shot learning approaches, which are being adopted for federated learning. Li and Wang [32] applied transfer learning and model distillation for federated learning. Guha et al. [33] proposed one-shot federated learning for supervised and semi-supervised setups for learning global models in one round of communication.

The existing literature shows that it is required to enable FL to learn from unlabeled data. One major limitation of the current approach is the high computation necessary for the client, for example, retraining a model under the transfer learning approach or inferring labels using the model inference, which are all computationally intensive. Many of these approaches depend heavily on approximation, which can propagate errors to the global model. The proposed work uses simple clustering-based labeling, which requires lower computation. The expand phase exploits the drawback of centralized clustering, i.e., to obtain good clustering performance, the model may result in a higher number of clusters (by splitting similar items into different clusters to improve upon cluster density or other metrics). We aim to group similar items independent of the number of total clusters because during the shrink phase, the clusters will be mapped to the number of the required class.

## 3. Expand and Shrink: Federated Learning with Unlabeled Data Using Clustering

### 3.1. Problem Definition

Data labeling is necessary for supervised learning and takes significant time, effort, and resources (computational and financial). Data labeling methods can be divided into two main groups: manual and automatic. The human annotators perform the fully manual labeling, and the programs perform fully automatic labeling. However, manual and automatic can assist each other; for example, if human annotators assist in automatic labeling, it is called human in the loop (HITL). Besides labeling, humans also play the role of verifier or reviewer for the data labeled by other annotators. Most of the time, a human annotator also supervises automatic labeling and acts as a verifier. Human labeling is costly and time-consuming. However, it provided better-quality labeled data. Many applications only considered human labeling; for example, only labels from medically trained professionals are acceptable for medical-related models, such as cancer cell or tumor classification.

The FL system can label data at the client device with or without client participation for labeling and label verification. Client participation in labeling and verification can be implicit, like behavior-based auto labeling, i.e., using the “client click” on the advertisement as a class label or client acceptance of text suggestion as a class label. Such an implicit approach can also be termed as labeling automation without assistance. In explicit participation, the client must actively engage with unwanted and unfeasible labeling processes. Due to client participation, it is called labeling automation with assistance (the item and label are auto-generated, and the user has to verify).

The proposed work, Expand and Shrink, does not require user participation for labeling or verification and is fully automated. The proposed work adopts the popular cluster-then-label approach. In the expand phase, we apply a clustering algorithm to all unlabeled data. During the shrink phase, we use the truth dataset to map the resulting clusters to a specific class label (the possibility of many clusters being mapped to a single class label). With the expand step, we moved from the critical assumption of the cluster to class mapping approaches such that one cluster exactly corresponds to one class because in the proposed work, expand results in more clusters than classes.

**Figure 1:**
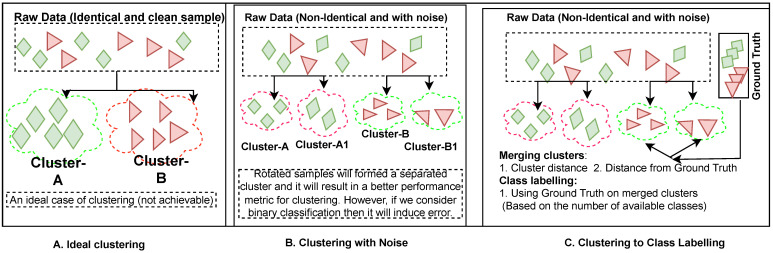
Overview of Expand and Shrink: (**A**) Ideal case of clustering, i.e., a set of data points are identical and form ideal clusters. (**B**) Expand step: Clustering is applied assuming variance in data points within a set and a higher number of clusters formed considering lower inertia. (**C**) Shrink step: Clusters are mapped to class labels using the truth label set and distance (one or more clusters can be mapped to a single class).

Figure 1 presents the proposed Expand and Shrink approach. The expand phase is based on a “higher number of clusters decreases the inertia and lower inertia is better”. So, with a threshold (*I*), we keep increasing the number of clusters and stop when the inertia value comes under *I*.

Figure 1A shows the ideal case of clustering, where the data point has a proper and uniform shape, resulting in two suitable clusters. However, such a perfect case is rare, and often in the real world, we have scenarios where data points come with variance and may result in different clusters as shown in Figure 1B. The expand phase considers this real-world use case and so tries to obtain the maximum clusters by grouping the data points with variance into different clusters. Figure 1C shows the shrink step, where the clusters are mapped to the class label (the number of classes decided as per the selected supervised learning task, for example, 0–10 for MNIST [15]) by using the truth label set based on the distance and seed sample (labeled sample is mixed with unlabeled data before clustering).

### 3.2. System Model

**Figure 2:**
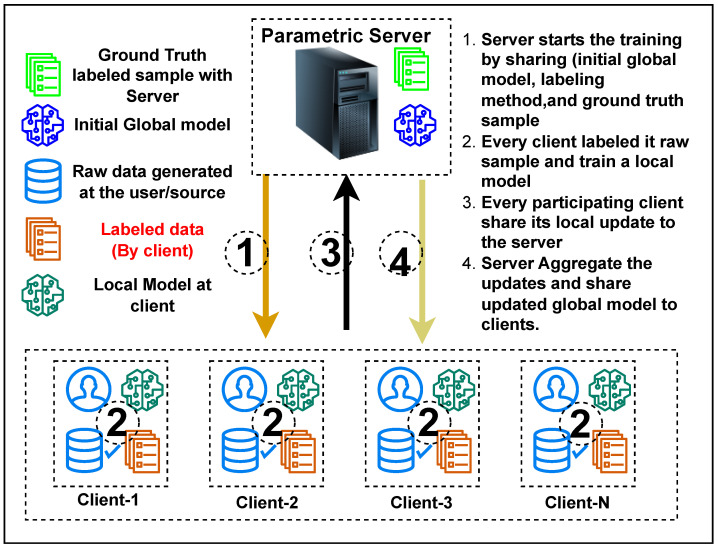
Steps of federated learning with data labeling process at the client device.

Providing class labels to the data at the client device must be automated and performed on the device. Semi-supervised approaches are adopted in federated learning for labeling the client’s data. We propose a clustering-based approach to label the data sample at the client machine in a federated learning architecture. Figure 2 shows the integration of the federated learning (FL) approach with the data labeling process on the client device. Table 1 lists the symbols and notations used in this paper.

The sets of *N* total and *S* selected participating nodes for each round are defined, respectively, as N={1,2…,N} and S={1,2,…,S}, where N=|N|, S=|S|, and S<N.

$U_{i}$ is a set of local unlabeled data of client *i*, where the client *i* initially has a set of $n_{i}$ number of unlabeled training samples client i∈N denoted as $U_{i}=\left\{x_{i}^{1},x_{i}^{2},\ldots ,x_{i}^{n_{i}}\right\}$. After applying to the Expand and Shrink method by the client, each data sample of client *i*$x_{i}\in U_{i}$ will be mapped to the class label in the set of class label *Y* as follows:

$$\;x_{i}\mapsto y_{i},$$

where $y_{i}$ equals g∈{1,2,…,G}, which is an element of a set of class labels denoted as Y. The local labeling gives mapping results in a set of elements $D_{i}=\{\left(x_{i}^{1},y_{i}^{1}\right),\left(x_{i}^{2},y_{i}^{2}\right),\ldots ,$$\left(x_{i}^{n_{i}},y_{i}^{n_{i}})\right\}$. *D* represents the labeled dataset from all selected clients denoted as $D≔\{D_{1},D_{2},\cdots ,D_{S}\}$. Each element in the dataset $D_{i}$ is a pair $\left(x_{i},y_{i}\right)$, where $x_{i}$ represents a data point and $y_{i}\in Y$ is the corresponding class label. We introduce a policy set P, which is the policy vector of the federation that is sent to every node by the parameter server. It has a set of values as P={M,V,C}:

- Model (*M*): Supervised learning model selected by the parameter server to train using federated learning, for example, CNN, LSTM, etc.
- Validation dataset (*V*): A small proportion of the labeled datasets are provided to each client for the shrink phase of labeling.
- Clustering method (*C*): The client can choose a set of clustering methods. We are currently using only K-mean clustering.

### 3.3. Data Labeling with Expand and Shrink

For federated learning, each node/device has to join the federation. The joining process will start with the initial setup, i.e., the parameter server will share the P with the client, and each client will perform the data labeling by executing the Expand and Shrink method. The client will be selected for a training round based on the ”labeling status“ along with other existing selecting criteria, such as power and computation availability, etc.

We use modified *K*-means clustering to perform data labeling for each client. The data labeling process is independent of the federated training process, and the client joins the federation and obtains the P for performing the labeling of its unlabeled local data $U_{i}$. The federated learning process will be similar to the existing approach.

**Step 1—Join:**  A device joins the federation and obtains policy vector P from the parameter server.
**Step 2—Expand:**  Each client applies clustering to its unlabeled data and uses the inertia versus the number of clusters to find the value of *K*, where *K* is the total number of clusters that give the best inertia value. The inertia represents the sum of squared distances between each data point and its assigned centroid. Any clustering algorithms can be used, as in the proposed work was evaluated using *K*-means. Thus, the objective function of the *K*-means clustering is defined as a minimization problem, and it is presented in Equation (1):
  (1) $$\underset{S}{\mathrm{argmin}}=\sum_{k=1}^{K}\sum_{x_{i}\in S_{k}}{∥x_{i}-\mu_{k}∥}^{2}$$
  where $x_{i}$ represents the sample of client *i*, $S_{k}$ is the set of clients belonging to cluster *k*, and $\mu_{k}$ is the centroid of cluster *k*.
**Step 3—Shrink:**  The client with *K* number of clusters starts to shrink by using the distance between the clusters and the sample in validation dataset *V* shared by the parameter server. The distance calculation will create S using Equation (3), which is the K×G score value matrix, where *K* is the total number of clusters in the expansion step and *G* is the total number of classes for the supervised learning task. In S, each row will have the distance score *s* of a cluster k={1,2,…,K} against all the classes, i.e., 1,2,…G. The distance matrix S is constructed as
  (2) $$S=\left(\begin{array}{cccc} s_{1,1} & s_{1,2} & \cdots & s_{1,G}\\ s_{2,1} & s_{2,2} & \cdots & s_{2,G}\\ \vdots & \vdots & \ddots & \vdots \\ s_{k,1} & s_{k,2} & \cdots & s_{k,G} \end{array}\right)$$
  Each distance score can be calculated using the Euclidean distance, computed as
  (3) $$s_{r,c}=\sqrt{\sum_{i=1}^{n}{\left(c_{i}-v_{i}\right)}^{2}}$$
  where *c* and *v* are vectors for the centroid and sample in the validation set for the respective clusters and class labels. So, $c_{i}$ and $v_{i}$ represent the corresponding elements in the vectors *c* and *v* at the same index *i*.
  For merging clusters, we need to assign one or zero based on distance. The cell with the minimum value in each row will be marked as 1, indicating the cluster close enough to a particular class. In summary, each row of S will be converted to $S^{\prime }$ as
  (4) $$s_{r,c}^{\prime }=\left\{\begin{array}{cc} 1, & \mathrm{if}\;s_{r,c}=\min\left(S_{k}\right)\mathrm{for}\;\mathrm{row}\;k\\ 0, & \mathrm{otherwise} \end{array}\right.$$
  where $s_{r,c}$ represents an element of the distance matrix on the row r={1,2,…,K} cluster index and the column c={1,2,…,K} class index.
  Now, for merging cluster(s), each column vector will be scanned for 1, and the respective cluster *k* will merge as
  (5) $$D_{c}=\underset{k\in K|s_{r,c}^{\prime }=1}{⋃}k$$
  Then, each member of the respective cluster will be merged as one larger cluster, and it will be labeled as per the respective column class as
  (6) $$D_{i}=\underset{c\in Y}{⋃}D_{c}$$
  Merging clusters and labeling with respective class labels will create labeled data of each client *i*.
**Step 4—Ready State:**  The client can set its status to *ready* after completing the data labeling so the server can use this information while selecting the client for training.

**Figure 3:**
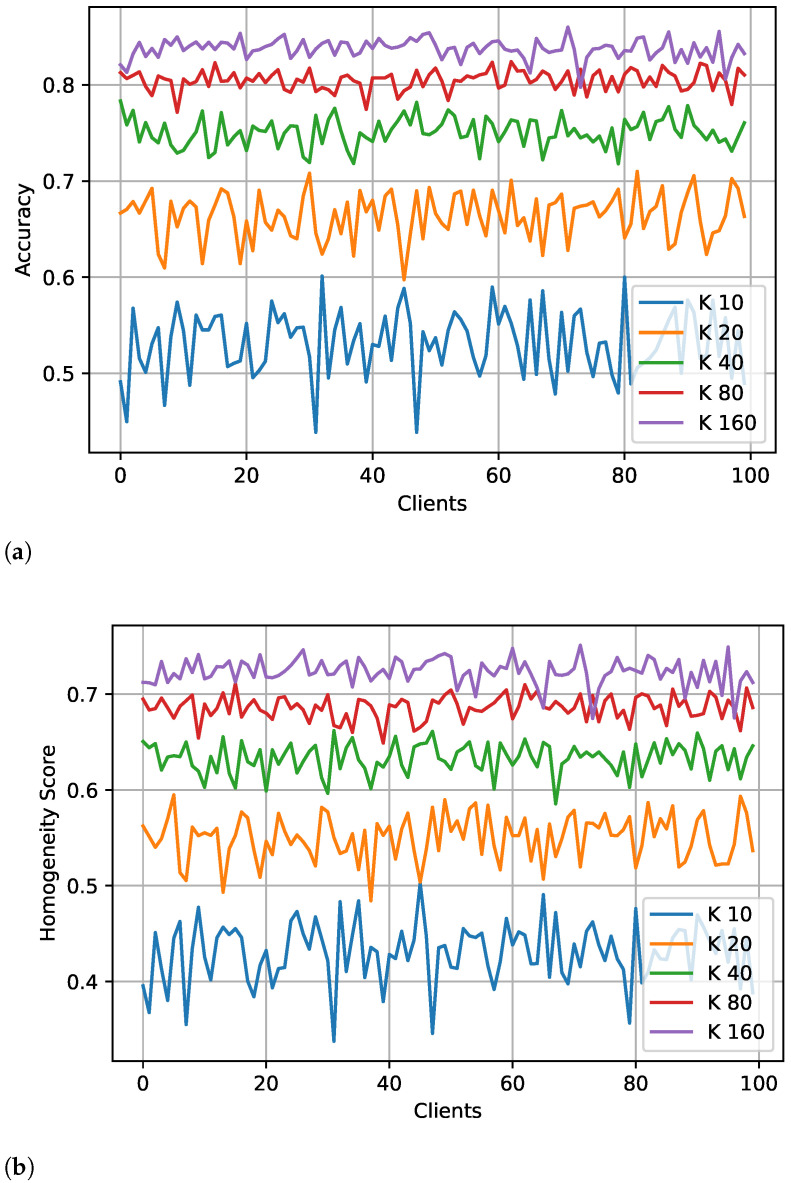

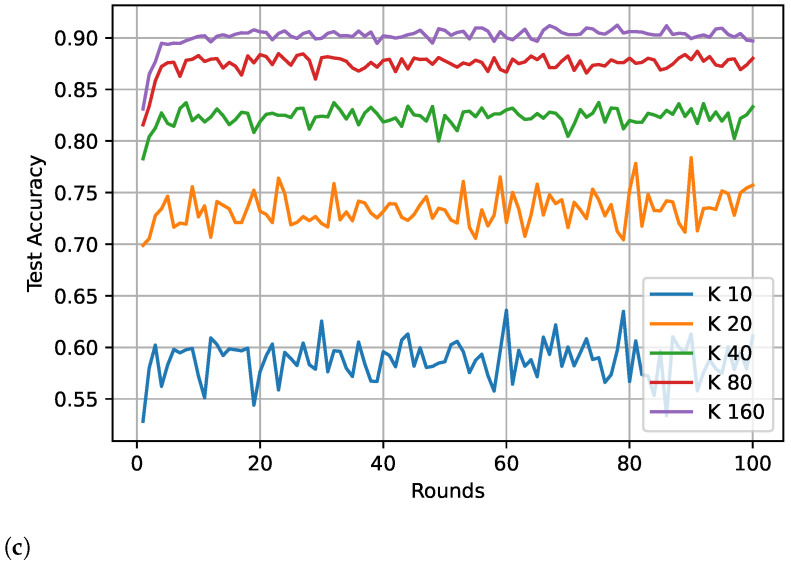
Performance of data labeling without training using a varying number of clusters (**a**,**b**), and global test accuracy under different rounds of training (**c**). (**a**) Accuracy of local client data (unlabeled) after labeling by the proposed method; (**b**) Homogeneity score of client data (unlabeled) after labeling by the proposed method; (**c**) Global test accuracy.

Figure 3 shows the result of labeling in terms of the accuracy and homogeneity score (Figure 3b), i.e., the outcome of the proposed algorithm on the unlabeled dataset of an individual client without training (Figure 3a) and global test accuracy after labeling with a varying number of clusters and training rounds (Figure 3c). The data labeling accuracy shown in Figure 3a is 85–90%, equivalent to human-level accuracy, considering the labeling errors in various datasets mentioned by Northcutt et al. [34]. The labeling performance in terms of accuracy and homogeneity score has less variation by increasing the clients, and the value of both metrics also improves with high clusters in the expand phase. Due to the larger client participation, each has fewer samples, which offers another benefit, and the proposed method works with smaller datasets, which is often the case in FL. A similar trend is observed in global test accuracy with different numbers of clusters and training rounds. The global accuracy gets stable with the higher number of clusters, while the number of training rounds has a smaller impact, so we can stop training with an early stop. We evaluated the labeling result using the truth label available for the experimental dataset. However, measuring only the accuracy of labeling will not be possible in a real-world scenario because there will be no true label. Once the client completes the data labeling process using the proposed method (Algorithm 1), any FL approach can be applied to the labeled data without modifying the existing approach. However, the labeling process must be integrated with the overall training steps as shown in Figure 2. Further, each step of FL with unlabeled data is explained in detail, and Algorithm 2 presents the pseudocode of the overall training.
**Algorithm 1** Data labeling at each client using Expand and Shrink.**Require:** *U*: Set of unlabeled data, *V*: validation dataset**Ensure:** *D* ($x_{i}\mapsto y_{i}$) for 1≤i≤N     {**Expand:** Create *K* clusters using a clustering algorithm}1: K← Expand(*U*) using Equation (1)2: S←s(c,v) using Equations (2) and (3)     {Find the minimum score in each row, mark it with 1 and the others with 0}3: $s_{r,c}$←$\left\{\begin{array}{cc} 1, & \mathrm{if}\;s_{r,c}=min\left(S_{k}\right)\;\mathrm{for}\;\mathrm{row}\;k,\\ 0, & \mathrm{otherwise}. \end{array}\right.$     {**Shrink:** Merge clusters}4: $D_{c}\leftarrow {⋃}_{k\in K|s_{r,c}^{\prime }=1}k$5: $D_{i}\leftarrow {⋃}_{c\in Y}D_{c}$6: **return** $D_{i}$

**Step 0—Data Labeling:**  A new client joins the federation and obtains P from the parameter server and labels its unlabeled data independently and free from the training round. After labeling, the client changes its status to ready.
**Step 1—Initialization:**  The parameter server selects *s* number of clients from N for federated training, initializes the global model *M* and shares it along with the validation dataset, i.e., *V* with each selected node in S. The *V* is a set of labeled pairs of (x,y).
**Step 2—Local Training:**  Each client applies Algorithm 1 on its local unlabeled samples *U* (explained in the previous section). Each node trains the model (*M*) on its self-labeled dataset and calculates the gradient difference using Stochastic Gradient Descent (SGD).
**Step 3—Client Update Sharing:**  Each node shares the calculated gradient difference ($\Delta \theta_{i}$) with the parameter server. The gradient is calculated by applying local training on θ using $D_{i}$.
**Step 4—Global Aggregation:**  For each global training round, the server collects and aggregates updates from each participating client ($N_{i}\in S$) and updates the previous model ($\theta \leftarrow \theta -\eta \cdot \Delta \theta_{\mathrm{avg}}$).
**Step 5—Updated Global Model Sharing:**  The final updated global model is shared with previously participating clients, and if the updated model is shared with new participants, then this step is similar to step 1. So, this step is optional and depends upon the training policy.

Steps 1–5 are performed for one training round of federated learning, and a single model is trained in multiple rounds. The termination criteria for training can be a combination of different requirements, such as desired accuracy, the maximum allowed training time, data available, etc. The following section presents the experimental setup and results of the experiments.
**Algorithm 2** Federated learning using Expand and Shrink on unlabeled data.**Require:** U: Unlabeled Data, *N*: Number of clients, *E*: Number of communication rounds   1:Initialize P, i.e., global model M as θ, *V*, and *C*   2:**for** each round *r* from 1 to *E* **do**   3:    Randomly select a subset of clients S    // Local Model Update   4:    **for** each client *i* in S **do**   5:         Receive P from the server   6:         $D_{i}\leftarrow$ Run Expand and Shrink on $U_{i}$ using Algorithm 1   7:         $B_{i}\leftarrow$ Split $D_{i}$ into batches for client *i*   8:         **for** each local epoch **do**   9:             Compute local update: $\Delta \theta_{i}\leftarrow \mathrm{ClientUpdate}\left(B_{i},\theta \right)$   10:        **end for**   11:        Send local update $\Delta \theta_{i}$ to the server   12:     **end for**     // Server Aggregation and Global Model Update   13:     Server aggregates the local updates:   14:     $\Delta \theta_{\mathrm{avg}}\leftarrow \frac{1}{|D_{i}|}\sum_{N_{i}\in S}\Delta \theta_{i}$   15:     Update global model: $\theta \leftarrow \theta -\eta \cdot \Delta \theta_{\mathrm{avg}}$   16:**end for**   17:**return** θ

## 4. Experiments and Result

### 4.1. Experimental Setup

We performed all the experiments on a server computer with Intel(R) Core(TM) i9-10980XE CPU @ 3.00 GHz processor, 251GB RAM, and 2xNVIDIA GeForce RTX 3090, and the same computing resources were used for running labeling code at client and federated learning. At the software end, we used the Ubuntu 18.04 64-bit operating system and Python 3.9 with different modules and frameworks, such as TensorFlow2, TensorFlow Federated (TFF), and Keras3, to implement the labeling scripts and federated learning.

During the implementation of the proposed work, we conducted all experiments with a fixed batch size of 64 (step 7 in Algorithm 2), which is standard practice. We followed the literature for model selection and considered using different models per the dataset. So, for MNIST [15] and FMNIST [35], we used a simple two-layer (Linear-ReLU-Linear-ReLU-Linear) neural network (TwoNN). Similarly, we trained the ResNet9 for CIFAR10 [36]. We used CrossEntropyLoss() as a loss function with the SGD optimizer, and in all the experiments, we used FedAvg() as an aggregation algorithm (step 14 in Algorithm 2).

### 4.2. Dataset Preparation

We validated the proposed work with experiments on MNIST datasets [15] and performed training and testing on FMNIST [35] and CIFAR10 [36] for comparison with similar works from the literature. In centralized training, 5% of the training data is used as a truth set while using the same sample numbers as in the default test set, i.e., 1000. After keeping 5% of the training data as a truth set for federated learning, the rest of the training samples were divided into 100 clients. The division of remaining samples among clients was randomized after properly shuffling the sample and sharing the required percentage with each client.

**Figure 4:**
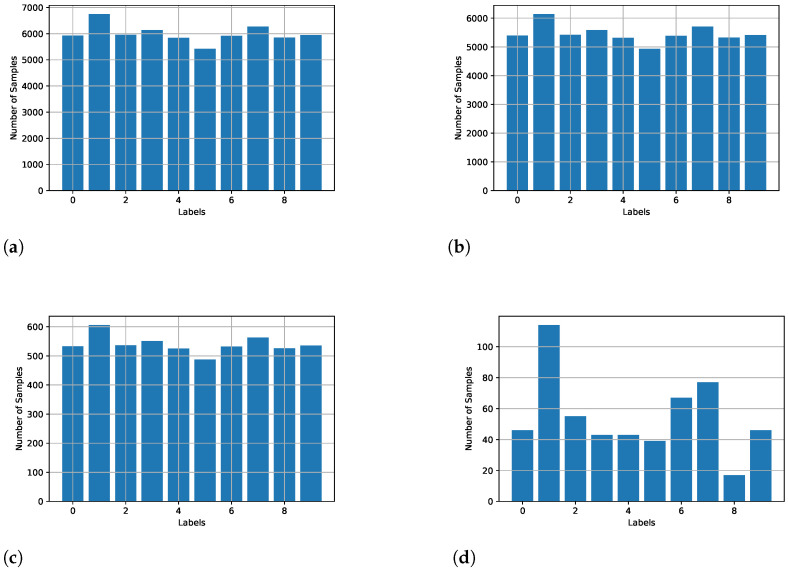
Sample distribution at each client. In figure, (**a**) Data samples per class in the dataset; (**b**) Training data samples per class; (**c**) Truth data samples per class; (**d**) Single client unlabeled data samples per class.

The experiments in the proposed work are limited to the iid case, i.e., each client receives an equal percentage of training data while keeping the same sample ratio for each class. Further, Figure 4a–c show the sample distribution per class in the overall dataset, training, and test dataset, respectively. Figure 4d shows the sample distribution per class for a random client. We only considered the iid use-case. However, the number of samples per class is not uniform for each client, so our experiment has elements of non-iid but not strictly non-iid.

### 4.3. Labeling and Training Time

**Figure 5:**
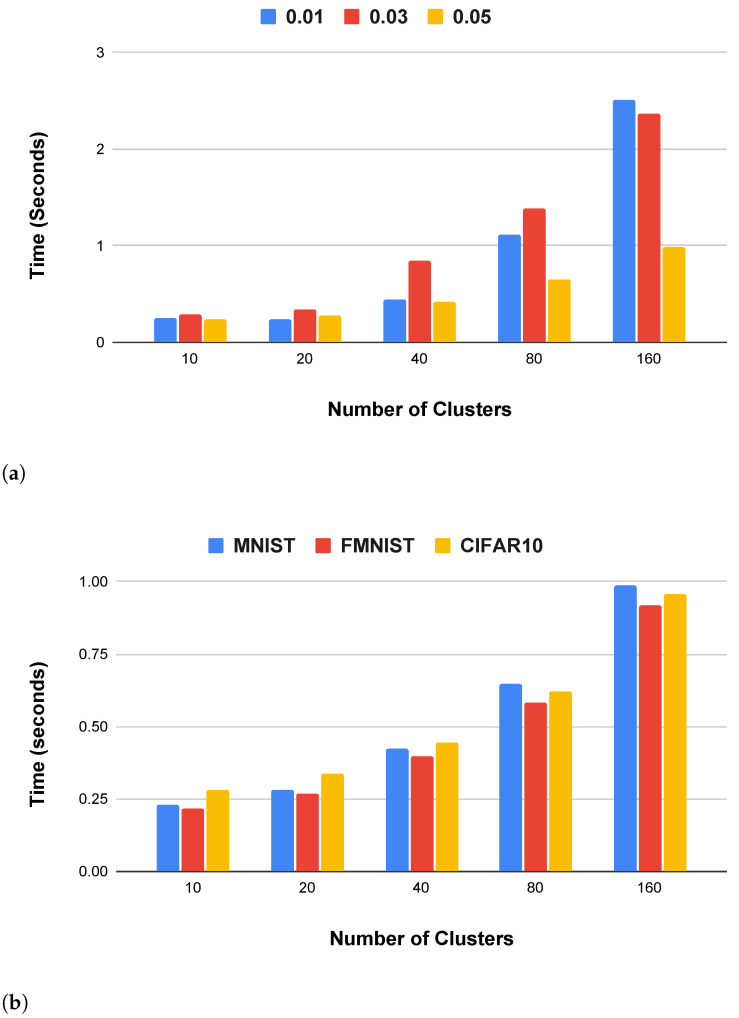
Labeling time by the proposed Expand and Shrink method. In figure, (**a**) Labeling time for MNIST with different ratios of the truth set; (**b**) Labeling time with 0.05 truth set.

The proposed method aims to be computation-effective in terms of time for performing labeling at the client. We experimented to understand the required labeling time with a different ratio of the truth label set and varied clusters for all three datasets. Figure 5 shows the labeling time taken by the proposed method. Figure 5a shows the labeling time only for MNIST under three ratios of truth label, i.e., (0.01, 0.03, and 0.05), while Figure 5b shows the time only for truth label ratio 0.05 for all three datasets. The result shows that the average labeling time for all 100 clients is below 3 s. Interestingly, a higher truth label ratio reduced the labeling time, and this is because the number of samples in each client gets reduced, and hence, the clustering takes less time. From Figure 5b, we can observe that the labeling time is independent of the data type (CIFAR10 has color images, while MNIST is grayscale) and a higher numbers of clusters will increase the labeling time, which is obvious.

**Figure 6:**
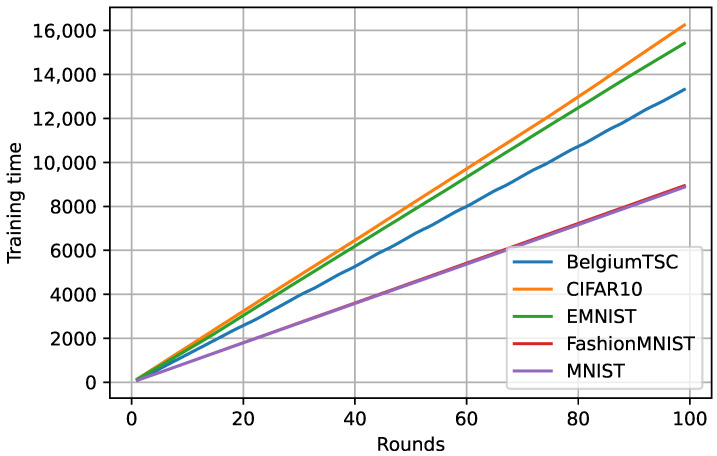
Training time of the proposed method on various datasets.

We also calculated the training time of the proposed method on various datasets. From Figure 6, we can observe that the training time is consistent with the number of rounds and size of the dataset, while it is varying as per the dataset type of sample. For example, MNIST and FashionMNIST are the same size and have grayscale images; in contrast, the other three datasets are larger and have color images.

### 4.4. Expand and Shrink: Centralized Learning

We first validated the proposed Expand and Shrink labeling in a centralized learning setting. Later, in the discussion section, we presented observations, comparisons, and results for centralized and FL setups. Although centralized learning differs from the federated setup, we will use this setting to demonstrate the general behaviors of the algorithms. Some key differences are that all unlabeled data are available at once in centralized learning, and more samples are available. At the same time, in FL, each client will have a lower number of samples. In a non-iid setup, each client will also have a different number of samples for each class that will further induce complexity. Table 2 shows the accuracy of the final model in centralized learning training after providing labels to the training sample using the proposed algorithm. The experimental results are shown for different numbers of clusters created (10, 20, 40, 80, and 160) in the expand step and for different ratios (0.01, 0.03, 0.05, 0.07, and 0.09) of data used from the validation or truth set.

**Figure 7:**
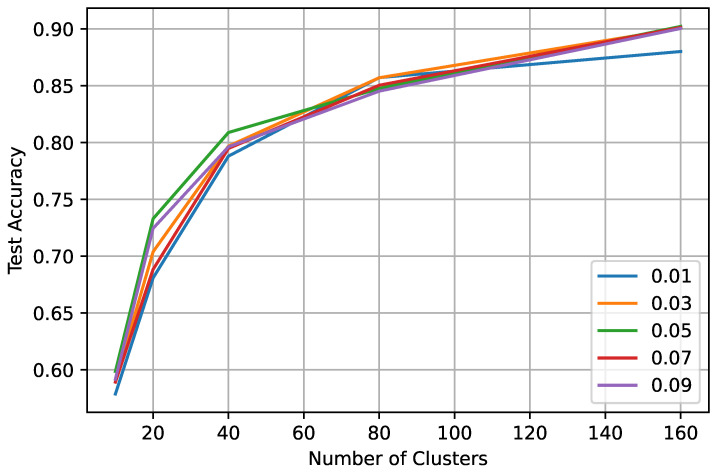
Test accuracy in centralized settings on a varying truth label ratio between 0.01 and 0.09.

In the centralized scenario, the best accuracy is gained with a minimum of 0.03 truth-labeled data and 160 clusters. Using a higher ratio than 0.03 of the truth label has a minimal effect on model performance. The results in Table 2 further verify our assumption that increasing clusters during the expand phase results in better labeling and increases the model’s accuracy. Figure 7 shows the test accuracy of the model in centralized learning with different configurations, and we can observe that the accuracy increases with the number of clusters.

### 4.5. Expand and Shrink: Federated Learning

**Figure 8:**
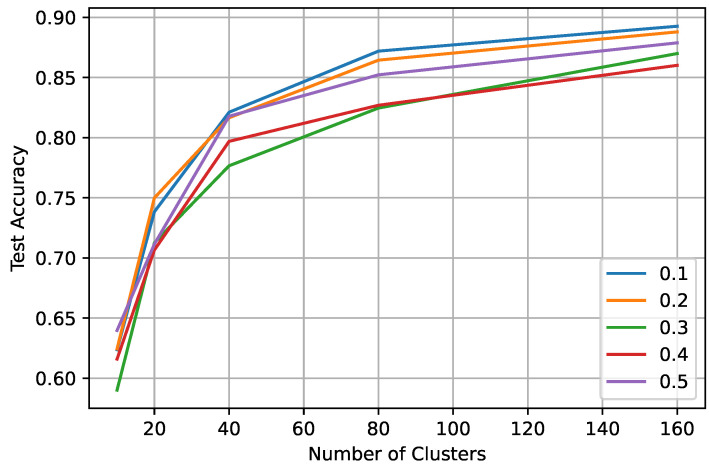
Test accuracy in federated settings on a varying client ratio between 0.1 and 0.5 with having a truth label ratio of 0.01.

**Figure 9:**
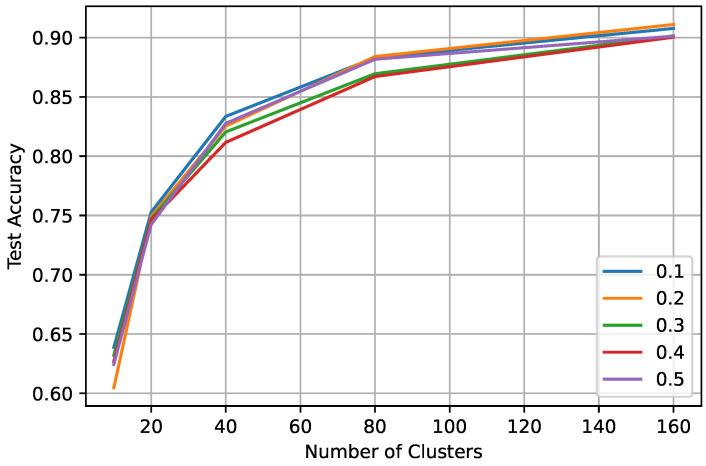
Test accuracy in federated settings on a varying client ratio between 0.1 and 0.5 with having a truth label ratio of 0.03.

In federated learning, we tested different scenarios by changing the number of clients participating in each round (0.1, 0.2, 0.3, 0.4, and 0.5 of 100 clients), percentage of validation, or truth set (0.01 and 0.03) for the iid datasets. Table 3 shows the test accuracy of FedAvg with the proposed algorithm for 0.01 and 0.03 validation/truth datasets for a different ratio of participating clients and the varying number of clusters in the expansion stage of the labeling process. With only 0.01 of truth label and 10 clients, the global model obtained 89% test accuracy. From Table 3, we can observe that increasing truth labels from 0.01 to 0.03 only improves the performance by 1–2%. However, with either ratio of the truth labels, the FL model can achieve the same test accuracy as the centralized model. In FL learning, the model performance increases with the number of clusters (Figure 8 and Figure 9), and the performance varies slightly (±1–2%) with the increasing number of client participants. However, more client participants delay the convergence, resulting in more communication rounds. So, it is suggested to train the FL model with a lower number of clients participating in each round. We can also observe that after labeling at the client (Figure 3), the highest accuracy of 80–84% is achieved, while after training the global model, an accuracy of 0.91 is achieved, showing an improvement of over 0.10.

### 4.6. Comparison with Existing Work

We also compared the proposed work with existing works. FedUL [25] and FedMatch [20] also performed data labeling on the client side for FL. FedUL uses the class prior probability instead of any labeled dataset as a truth or validation set. The proposed work is similar to FedUL in considering that the client has unlabeled data, and labeling is fully automated at the client. However, the global test accuracy for the MNIST dataset [15] is not mentioned, so the comparison is not possible due to the difference in performance metrics.

In FedMatch, there are two scenarios: labels-at-client (client has labeled data) and labels-at-server (only server has labeled data). Although the server sent the labeled dataset to all clients in the proposed work, it is similar to labels-at-client because labeling is only performed at the client. Although our approach is similar to the labels-at-client approach, the experimental dataset differs in the proposed work and FedMatch [20].

Following the experimental details of FedMatch, we extended our experiments and adopted similar deep learning architecture, i.e., ResNet-9 and the CIFAR-10 dataset [36], along with other FL-related configurations like the number of clients and client participation percentage. Table 4 shows the performance of the proposed work on CIFAR-10 [36] with two levels of the truth dataset, i.e., 0.01 and 0.003.

We obtained the best accuracy rates of 91% and 79% for MNIST and FMNIST with 0.03% of the truth label and 0.1% and 0.2% of the client participation, respectively. Similarly, the best accuracy for CIFAR10 is 30% with 0.03% of truth label and 0.2% of the client participation. From the experimental result, we can observe that the highest number of clusters during labeling and the lowest number of client participation provides the best performance of all three datasets and models.

In the proposed work, we only used 600 labeled data, while FedMatch uses 5000. However, the proposed work and FedMatch differ in the way of sharing the truth set. We share the same truth set among all the clients, while Fedmatch shares a unique labeled set with each client. FedMatch achieved a global accuracy of 52.25% for the CIFAR-10 dataset [36] in non-iid with 100 clients. With the ResNet architecture, we achieved 46% accuracy with 50% client sharing and only 5% labeled truth set. Interestingly, with the ExpandShrink, our labeling accuracy was 76%. It is also to note that the proposed work performed training after labeling all the samples at the client side, while FedMatch training was performed with labeled and unlabeled data.

The complexity comparison of the proposed and existing work can be made in two aspects: (1) the computational requirement for implementing the labeling method, and (2) the time and space requirement for training and getting the model from the federated learning process. FedUL [25] uses class-conditional distributions to provide surrogate labels to training data, and the model is trained on the surrogate-labeled dataset, and later, the wanted model is recovered. So, the computational cost should be considered two times; however, the proposed work uses extra computation for labeling, and there is no computation overhead for model training. Similarly, FedMatch [20] uses inter-client consistency loss to train with labeled and unlabeled data. However, generating, searching, and sharing helper agents increase the computation overhead compared to the proposed work.

## 5. Conclusions

The data labeling at the source device is critical for the practical use and adaptability of FL. The proposed method addresses the issue of the unlabeled dataset for supervised federated learning. In addition, the proposed method makes it possible to train a supervised model in federated learning without labeled data at the client device, enabling extensive data availability for training. With the proposed data labeling method, the model performs similarly to unlabeled client data in terms of accuracy and training loss compared to the traditional FL with the labeled dataset. We achieved accuracy rates of 87% and 90% by using 0.01 and 0.03 truth labels, respectively. The trade-off between the computation cost of automatic data labeling at the client device and model performance would be favorable and acceptable given the need for labeling at the source device. Obtaining labeling from the client can increase the risk of model poison attacks because the server and other clients will have less control over the global model due to the decrease in the truth sample and the expected possibility of an increase in noise in the data. However, verifying such a trend is out of the scope of the proposed work and can be conducted as future work. In future work, we aim to experiment with *non-iid* data distribution and identify specific in-device data labeling challenges and their mitigation in IoT environments. 

## Figures and Tables

**Table 1:** List of symbols used for system definition in equations and algorithms.

Symbol	Description
*C*	Clustering method
$c_{k}$	Vector representation of centroid of cluster *k*
$D_{i}$	Set of the local dataset of client *i*
*E*	Total number of communication rounds
*g*	Index of the class label
*G*	Total number of classes
*i*	Client index
*j*	Round index
*k*	Cluster index
*K*	Total number of clusters (|K|)
$L_{i}$	Set of the local labeled dataset of client *i*
*M*	Learning model
$\mu_{k}$	Centroid of cluster *k*
*N*	Total number of clients (|N|)
$n_{i}$	number data samples of client *i*
P	Policy set
S	Distance matrix
$S^{\prime }$	Normalized (1/0) distance matrix
*S*	Total number of selected clients (|S|)
$S_{k}$	Set of clients belonging to cluster *k*
$s_{r,c}$	Element of distance matrix on row *r* and column *c*
$U_{i}$	Set of unlabeled dataset of client *i*
*V*	Truth set
$v_{i}$	coordinate of validation sample
$x_{i}$	Data sample of client *i*
Y	Set of class label

**Table 2:** Accuracy of centralized learning with all of the clusters in the expand step (10, 20, 40, 80, and 160) and a ratio (0.01, 0.03, 0.05, 0.07, and 0.09) of the truth label.

Ratio of Truth Label /Number of Clusters	0.01	0.03	0.05	0.07	0.09
10	57	58	59	58	59
20	68	70	73	68	72
40	78	79	80	79	79
80	85	85	84	85	84
160	88	90	90	90	90

**Table 3:** Accuracy with a different ratio of client participation (0.1, 0.2, 0.3, 0.4, and 0.5), the number of clusters (10, 20, 40, 80, and 160), and truth label ratio (0.01 and 0.03).

Ratio of Truth Label	0.01					0.03				
**Number of Clusters /Number of Clients**	**10**	**20**	**40**	**80**	**160**	**10**	**20**	**40**	**80**	**160**
0.1	62	73	82	87	89	63	75	83	88	90
0.2	62	74	81	86	88	60	74	82	88	91
0.3	59	71	77	82	87	63	74	82	86	90
0.4	61	70	79	82	86	62	74	81	86	90
0.5	63	71	81	85	87	62	74	82	88	90

**Table 4:** Accuracy of the proposed algorithm on multiple datasets to compare with existing works (L%: truth label ratio, NoC: number of clusters, C%: client participation ratio). Note: The value in bold represents the highest accuracy for the dataset.

L%		0.01					0.03				
**NoC /C%**	**Dataset**	**10**	**20**	**40**	**80**	**160**	**10**	**20**	**40**	**80**	**160**
0.1	MNIST	0.6237	0.7384	0.8211	0.8719	0.8926	0.6387	0.7528	0.8335	0.883	**0.9077**
	FMNIST	0.6194	0.6996	0.7222	0.7237	0.7617	0.6142	0.7001	0.7207	0.7461	**0.779**
	CIFAR10	0.2319	0.242	0.2478	0.2547	0.2663	0.2343	0.2486	0.2601	0.2706	0.2841
0.2	MNIST	0.6244	0.7499	0.8165	0.8644	0.8879	0.6047	0.7491	0.8253	0.884	**0.911**
	FMNIST	0.6002	0.7017	0.7165	0.7443	0.756	0.612	0.6982	0.7208	0.7477	0.7614
	CIFAR10	0.2283	0.2362	0.2535	0.2624	0.2662	0.2341	0.2551	0.2666	0.2674	**0.2963**
0.3	MNIST	0.5942	0.7126	0.7767	0.8246	0.8699	0.6321	0.7472	0.8202	0.8691	0.9015
	FMNIST	0.6164	0.6963	0.7198	0.7391	0.7622	0.606	0.695	0.7163	0.7394	0.7666
	CIFAR10	0.2244	0.2403	0.2501	0.2521	0.2617	0.2332	0.2478	0.2609	0.2765	0.2927
0.4	MNIST	0.6121	0.7066	0.7969	0.8269	0.8601	0.6271	0.7462	0.8116	0.8671	0.9003
	FMNIST	0.6191	0.6958	0.715	0.7305	0.761	0.6042	0.6974	0.7212	0.7372	0.7775
	CIFAR10	0.2304	0.2363	0.2421	0.2495	0.2662	0.2399	0.2549	0.2634	0.2741	0.2905
0.5	MNIST	0.6397	0.7115	0.8175	0.8522	0.8788	0.6245	0.7423	0.8276	0.8817	0.9012
	FMNIST	0.5977	0.6945	0.7184	0.7262	0.7635	0.5876	0.6981	0.7168	0.7381	0.7684
	CIFAR10	0.2331	0.2296	0.2456	0.2525	0.2762	0.2385	0.2533	0.267	0.2731	0.2843

## Data Availability

Data are contained within this article.

## Abbreviations

The following abbreviations are used in this manuscript:

AI	Artificial Intelligence
CNN	Convolutional Neural Network
DL	Deep Learning
FL	Federated Learning
FSSL	Federated semi-supervised Learning
HITL	Human in the Loop
IoT	Internet of Things
LSTM	Long Short-Term Memory
ML	Machine Learning
NN	Neural Network
non-IID	Not Independent and Identically Distributed
SGD	Stochastic Gradient Descent
SSFL	Semi-Supervised Federated Learning
SSL	Self-Supervised Learning
TFF	TensorFlow Federated
